# A Three-Dimensional Culture Model of Reversibly Quiescent Myogenic Cells

**DOI:** 10.1155/2019/7548160

**Published:** 2019-11-11

**Authors:** Salvatore Aguanno, Claudia Petrelli, Sara Di Siena, Luciana De Angelis, Manuela Pellegrini, Fabio Naro

**Affiliations:** ^1^Department of Anatomical, Histological, Forensic and Orthopedic Sciences, Sapienza University, Rome, Italy; ^2^Institute of Cell Biology and Neurobiology, CNR, Monterotondo, Rome, Italy

## Abstract

Satellite cells (SC) are the stem cells of skeletal muscles. They are quiescent in adult animals but resume proliferation to allow muscle hypertrophy or regeneration after injury. The mechanisms balancing quiescence, self-renewal, and differentiation of SC are difficult to analyze *in vivo* owing to their complexity and *in vitro* because the staminal character of SC is lost when they are removed from the niche and is not adequately reproduced in the culture models currently available. To overcome these difficulties, we set up a culture model of the myogenic C2C12 cell line in suspension. When C2C12 cells are cultured in suspension, they enter a state of quiescence and form three-dimensional aggregates (myospheres) that produce the extracellular matrix and express markers of quiescent SC. In the initial phase of culture, a portion of the cells fuses in syncytia and abandons the myospheres. The remaining cells are mononucleated and quiescent but resume proliferation and differentiation when plated in a monolayer. The notch pathway controls the quiescent state of the cells as shown by the fact that its inhibition leads to the resumption of differentiation. Within this context, notch3 appears to play a central role in the activity of this pathway since the expression of notch1 declines soon after aggregation. In summary, the culture model of C2C12 in suspension may be used to study the cellular interactions of muscle stem cells and the pathways controlling SC quiescence entrance and maintenance.

## 1. Introduction

Satellite cells (SC) lie between the basal lamina and the sarcolemma of skeletal muscle fibers. In adult muscle, they are quiescent until physical exercise or muscle damage induces their activation. SC proliferate and produce myoblasts that fuse either together or with existing myofibers, thereby allowing growth and repair of skeletal muscle [1]. A central question in adult stem cell biology regards the elucidation of the molecular mechanisms that preserve the regenerative potential of the tissues by maintaining a population of reversibly quiescent stem cells. Several findings suggest that reversible quiescence is not a state of cell inactivity but is the result of specific molecular programs [2, 3].

The study of SC biology *in vivo* is difficult owing to the complexity of the environment, the relatively low density of SC, and the absence of specific markers to recognize them [4]. It is not possible to maintain the quiescent state of SC *in vitro* because any isolation procedure triggers their activation and converts them into cycling myoblasts that undergo differentiation. SC lose their staminality because their return to quiescence is precluded in the monolayer culture by the lack of an appropriate niche.

The system of choice to characterize quiescent SC is to sort them by FACS from collagenase digested muscle. This procedure presents several problems since SC are heterogeneous and the antibodies chosen for FACS isolation select subsets of cells [5]. Furthermore, the isolation procedure activates SC, and their activation continues during FACS purification, altering the pattern of gene expression [6, 7]; for example, the mere detachment of the monolayer for routine subculture rapidly alters the expression of notch1, which plays a pivotal role in determining SC behaviour [7].

For all of these reasons, the development of culture systems that allow undisturbed reversibly quiescent myogenic cells to be studied is very appealing [4, 5].

Three-dimensional (3D) cultures of myogenic cells have already been employed to grow SC from primary cultures. When isolated cells are seeded in nonadherent dishes, they spontaneously form floating myospheres. Such cultures are performed in growth factor-rich synthetic media that induce the expansion of satellite stem cells and conserve their staminality. The comparison of published data suggests that different combinations of cells resulting from different isolation procedures and different culture media give rise to myospheres containing stem cells with different characteristics in terms of proliferation, marker expression, and differentiation capacity [8–15].

An important advantage of myospheres is that they retain cellular interaction, which allows the involvement of the notch pathway in satellite cell biology to be studied *in vitro*. In skeletal muscle, notch is a key regulator engaged in embryonic myogenesis in myoblast proliferation [16]. Around birth, it locates myogenic progenitors in the SC compartment under the basal lamina on muscle fiber [17], whereas in adult muscle, it maintains the reversibly quiescent state of SC [18, 19]. A reduced activity of the notch pathway determines the reduced ability of muscles in old animals to regenerate [20, 21]. Notch signalling is activated when a cell displaying a notch ligand (jagged1-2, dll1-3-4) on its membrane binds a neighbouring cell exposing a notch receptor (notch1-4).

The members of the hes/hey family of transcription factors mediate most of the notch response by forming heterodimers with MyoD that inactivate this factor, thereby blocking myogenic differentiation [22, 23]. In SC, different members of the notch family appear to cover different roles, although it is not clear how they elicit different cellular responses while sharing the same pathway. For example, notch1 is involved to a greater extent in proliferation during regeneration [24] while notch3 expression is highest in self-renewing SC [19, 25, 26].

To investigate whether 3D cultures may be used to maintain SC in a quiescent status and eventually use them in skeletal muscle regeneration, we adopted the murine C2C12 myoblast cell line.

We found that C2C12 cells in myospheres spontaneously downregulate notch1 and maintain the expression of notch3. The expression of hes/hey genes accompanying this pattern of activation is lower than that of C2C12 cells proliferating in a monolayer, but it is critical for the cells to remain quiescent and undifferentiated. Indeed, when notch signalling is chemically inhibited, the cells of myospheres exit quiescence and differentiate. These data on the level of activation of hes/hey genes in myospheres differ from those obtained in freshly isolated SC, whose hes/hey expression is higher during quiescence but declines when the cells proliferate. The possibility that the isolation of satellite cells alters notch activity highlights the advantage of using a culture system with cells readily available for analysis.

Within a wider context, we believe that the ductility of the niche that arises in the C2C12 model may shed light on how the addition of other cell types, such as fibroblasts and circulating immune cells, or of exogenous soluble factors and ECM components affects specific aspects of stem cell behaviour.

## 2. Results

### 2.1. Generation and Evolution of C2C12 Myospheres

**Figure 1:**
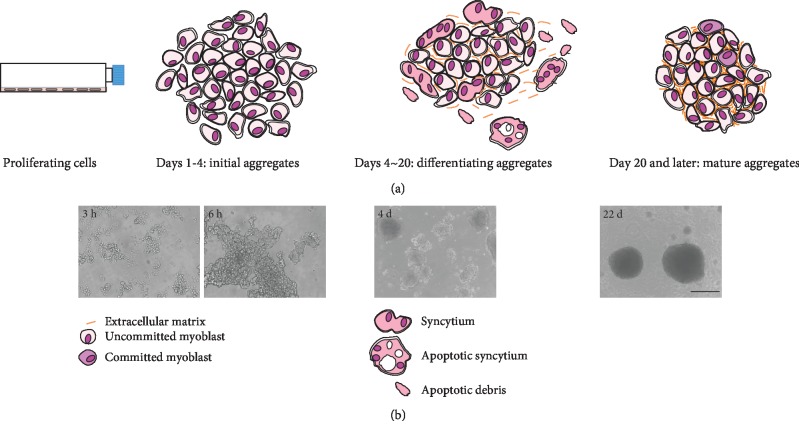
Scheme of myosphere evolution shown according to (a) culture time and (b) contrast phase microscopy.

Mouse C2C12 myoblasts seeded in nonadhesive conditions form aggregates that appear within 3-4 h. Initially, the cells join together in loose networks, which subsequently compact in spherical myospheres (Figure 1).

**Figure 2:**
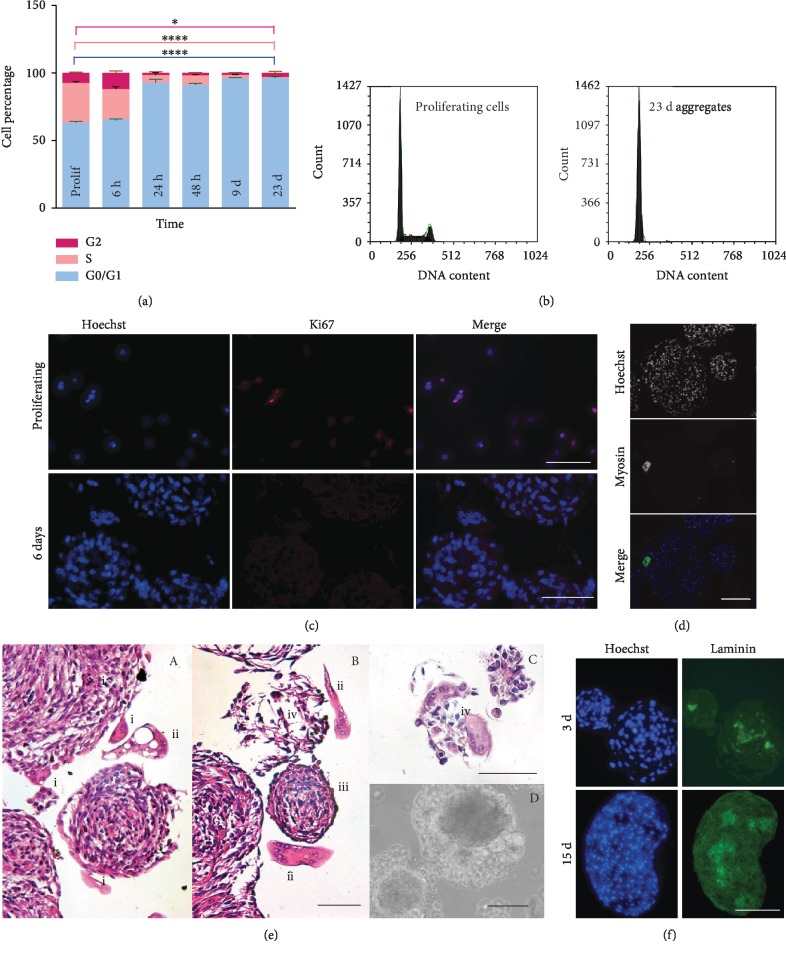
Characterization of C2C12 myospheres. (a) Bar chart from cytofluorometer data showing the proportion of cells (±SD) in different phases of the cycle, in a monolayer and in myospheres of different ages. ^∗^0.01 < *p* < 0.05 and ^∗∗∗∗^*p* < 0.0001. (b) Cytofluorometer plot comparing the cell cycle in cells proliferating in a monolayer and in 23-day myospheres. (c) Ki67 expression in a monolayer and 6-day myospheres. (d) Myosin expression evaluated by immunofluorescence. A labelled syncytium is at the periphery of a 9-day myosphere. (e) H&E staining of 9-day myospheres containing differentiating cells. Syncytia leaving the myospheres or internally located are indicated with (i), syncytia isolated in the medium after exit from a myosphere with (ii). (iii) indicates a mature myosphere that does not appear to contain syncytia. (e) in B and C, the symbol (iv) labels small myospheres dissolving after the detachment of syncytia. (f) Accumulation of extracellular matrix proteins in myospheres over time. Laminin expression in 3-day and 12-day myospheres. Scale bar: 50 *μ*m in (c), (d), and (f) and 100 *μ*m in (e).

At 6 h, flow cytometric analysis shows that the percentage of cells in G_1_, S, and G_2_/M in the aggregates is still the same as in the monolayer (Figure 2(a)). At 24 h, roughly 90% of the cells in the aggregates are in G0/G1; beyond 48 h, the percentage rises to 95-96%, remaining stable thereafter (Figures 2(a) and 2(b)). Evaluation of proliferation by the Ki67 expression (Figure 2(c), 6-day myospheres) confirmed the absence of proliferating cells in myospheres after 48 h of culture.

Between the 3rd and the 4th day of culture, some cells begin to fuse in syncytia. Sections of myospheres stained by haematoxylin and eosin or by immunofluorescence with an anti-sarcomeric myosin antibody display multinucleated syncytia located mainly in the periphery and often partially detached (Figure 2(e), C). At this stage, a halo that retains detached syncytia and degenerating cells appears around the myospheres (Figure 2(e), D). The halo can be easily removed by pipetting or by short DNase treatment, but it forms again in less than 24 h because the syncytia continue to leave the myospheres accompanied by mononuclear cells that form apoptotic bodies and release DNA once they are isolated in the culture medium (supplementary Fig. [Supplementary-material supplementary-material-1]).

The halo spontaneously disappears between the 18^th^ and the 25^th^ day of culture. The surface of myospheres becomes smooth and clean although histological sections occasionally display the presence of remaining syncytia that do not detach (mature myospheres, Figure 1, 22 d).

Mature myospheres have a diameter of 80-200 *μ*m and are formed by quiescent cells and an extracellular matrix, as shown by the presence of laminin which can already be detected by immunofluorescence 72-96 h after seeding and accumulates over time (Figure 2(f)). Mature myospheres kept in culture until 60 days do not display any further evolution except for a higher resistance to dissociation by proteases, which is evident at 33-35 days and makes it more difficult to isolate the cells. When differentiation terminates, the number of cells enclosed in the myospheres is 25.7 ± 3.7% of those initially seeded, as assessed in myospheres of 25 and 33 days after trypsinization and the cell count (*n* = 7). Besides the expulsion of syncytia, at least two other causes account for the loss of cells (Figure 2(e)). The first is that during the genesis of myospheres, a fraction of the cells does not aggregate and dies in the medium as a result of apoptosis (anoikis). The second is that when syncytia leave the myospheres, they frequently induce the disassembly of nearby mononuclear cells, which also degenerate (Figure 2(e), B and C; myospheres marked by (iv)). The cells in the myospheres are protected from apoptotic death, as demonstrated by the results of the TUNEL assay performed in the first 4 days of culture (not shown).

These data indicate that C2C12 cells aggregate when cultured in nonadherent conditions and that some of them differentiate, forming multinucleated syncytia that are lost.

### 2.2. Characterization of C2C12 Myospheres

**Figure 3:**
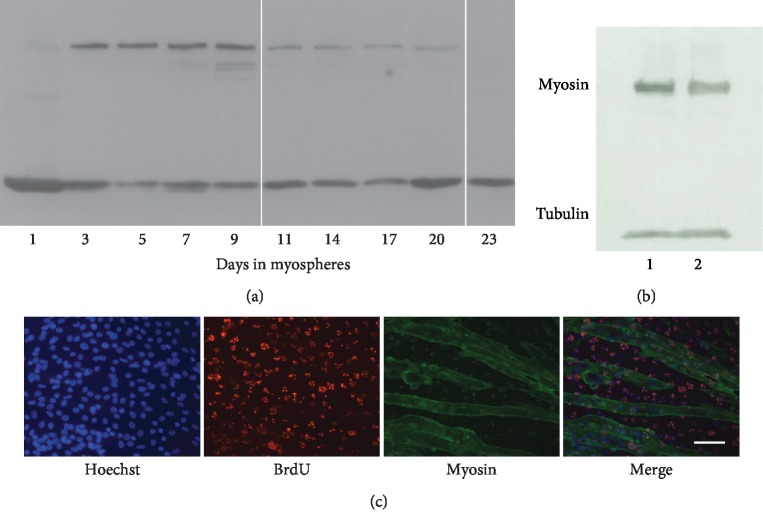
Trend of myogenic differentiation during myosphere maturation and differentiation potential retained in quiescent cells of mature myospheres. (a) Time course of myosin expression in myospheres by western blot from the 1st to 23rd day of culture. (b) The cells of mature myospheres retain the capacity to proliferate and differentiate. The western blot quantitatively compares myosin expression in a monolayer: (1) original C2C12 cells that were never grown as myospheres and (2) cells derived from the dissociation of 23-day myospheres plated in a monolayer and allowed to differentiate. (c) C2C12 cells dissociated from 23-day myospheres (mature); replated in a monolayer, the cells incorporate BrdU while proliferating before differentiating and expressing myosin. Scale bar: 50 *μ*m.

To investigate the rate of differentiation, we assessed the expression of myosin by western blot during the maturation phase of myospheres (Figure 3(a)). Myosin expression starts on the 3^rd^ day and peaks on the 9^th^-10^th^ day of culture. Thereafter, it gradually decreases until it becomes undetectable at 18-20 days in parallel with the loss of syncytia. If these mature myospheres are dissociated and the cells are plated in monolayer, they proliferate and then fuse, though to a lesser extent than the original C2C12 cells (Figures 3(b) and 3(c)). Indeed, the fusion index is 55.5 ± 2.6% in the original C2C12 cells and 16.7 ± 5.4% in myoblasts derived from mature myospheres (*n* = 6). Assuming that both cell types proliferate at the same rate after plating, this indicates that the ratio between C2C12 cells that conserve their differentiation capacity (stem cells) and those that cannot differentiate ranges from 1 : 1 at the beginning of the 3D culture to 1 : 5-1 : 9 in mature myospheres.

**Figure 4:**
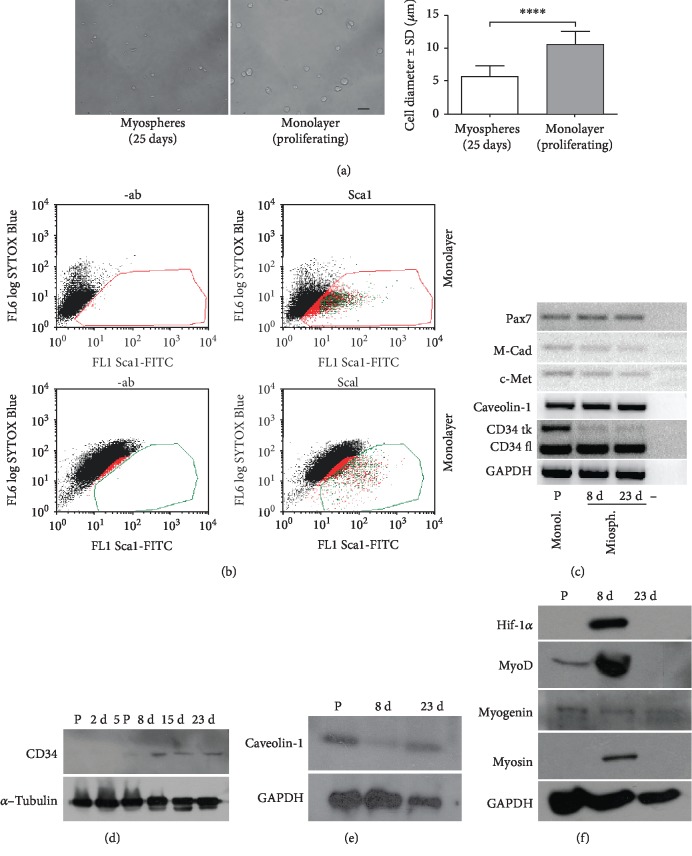
Cell size and marker expression. (a) Representative image comparing C2C12 cells shortly after dissociation of 25-day myospheres and detachment from the monolayer. The graph on the side shows the range of cell diameters. ^∗∗∗∗^*p* < 0.0001. (b) Flow cytometric profile of Sca1 expression in cells from myospheres (25 days) and from a monolayer. (c) Semiquantitative RT-PCR profile of common quiescent satellite cell markers in differentiating and mature myospheres. Myogenin and myosin indicate the presence of differentiating cells. P: proliferating cells in monolayer. (d) Time course of CD34 expression in myospheres by western blot. (e) Caveolin-1 expression in proliferating cells and in differentiating and mature myospheres by western blot. (f) Western blot comparing hif-1*α* expression in proliferating cells and myospheres, with the expression of myogenic markers.

Cells dissociated from mature myospheres are smaller than proliferating cells cultured in a monolayer (Figure 4(a)), which is in keeping with evidence showing that quiescent SC in vivo have few organelles in a little cytoplasm and a large nucleus-cytoplasmic ratio if compared with activated and proliferating cells [27, 28]. To assess the hypothesis that they might represent a model of quiescent SC cells, we evaluated the expression of different markers of SC. In particular, we estimated the expression of Sca1 because it is expressed in proliferating myogenic cells but not in quiescent cells [29].  Figure 4(b) compares the expression of Sca1 in C2C12 cells in myospheres with that of C2C12 cells in a monolayer as measured by flow cytometry. In agreement with previous results [29], C2C12 cells in myospheres are 1.3% Sca1^+^ whereas C2C12 cells proliferating in a monolayer are 30% Sca1^+^ positive.

Pax7 is a key marker widely used to recognize SC in skeletal muscle [30], though the fact that expression continues after myoblasts start proliferating limits the value of this factor for assessing quiescence.  Figure 4(c) shows that Pax7 is expressed in C2C12 cells in a monolayer and in myospheres at two different ages, thus suggesting that cells in the myosphere retain SC characteristics.

CD34 is a surface marker of various progenitor cells. It has been reported that a full-length transcript of CD34 is expressed in quiescent SC cells and that a truncated form is expressed in proliferating SC [31]. In myospheres, we only find the full-length form of CD34 mRNA which is expressed in quiescent SC *in vivo*, whereas proliferating C2C12 cells display both the truncated and the full-length forms of CD34 mRNA (Figure 4(c)), which give rise to the mature protein form (Figure 4(d)).

Caveolin-1 is expressed in satellite cells *in vivo* and is downregulated in activated satellite cells [32]. WB analysis of caveolin-1 expression in myospheres shows that it is upregulated in mature myospheres that contain quiescent cells (Figure 4(e)).

Figure 4(f) shows the expression of MyoD, myogenin, and myosin, which discloses the presence of activated cells (MyoD), cells committed to myogenesis (myogenin), and differentiated cells (myosin).

In summary, the generation of mature myospheres requires approximately 20 days, by which time one-third of the C2C12 cells initially plated retain their stem cell characteristics. Such cells express SC markers, are quiescent, and retain their differentiation capacity.

### 2.3. Hypoxia Is Not Involved in the Quiescence of Cells in Myospheres

Three-dimensional cultures are commonly used as a model of the solid tumour microenvironment and angiogenesis thanks also to the state of hypoxia harbouring in their internal layers. The hypoxic environment favours quiescence of various stem and precursor cell types, as shown by the viability of stem cells in post-mortem muscle [33]. Moreover, it has been shown that hif-1*α* and the notch intracellular domain (NICD) synergize to activate the expression of notch target genes, which inhibit the expression of myogenic factors in myogenic cells and maintain the stem cell state [34].

Based on these data and on the observation that in myospheres syncytia localize mostly in the more oxygenated external layers [35], we hypothesized a role of hif-1*α* in maintaining cell quiescence in the cells at the centre of mature aggregates. hif-1*α* was found in myospheres at 8 days of culture, when differentiating cells are present but was absent in later mature aggregates (Figure 4(f)).

This result is in keeping with a report by Ono et al., who found that myogenic cells express hif-1*α* during differentiation in normoxic conditions, in which hif 1 *α* plays an essential role as its silencing inhibits the differentiation of C2C12 cells [36].

This result indicates that the expression of hif-1*α* in myospheres is linked to myogenic differentiation and not to hypoxia and quiescence.

### 2.4. Notch Signalling Is Active in Myospheres and Is Required to Maintain the Quiescence of the Cells

**Figure 5:**
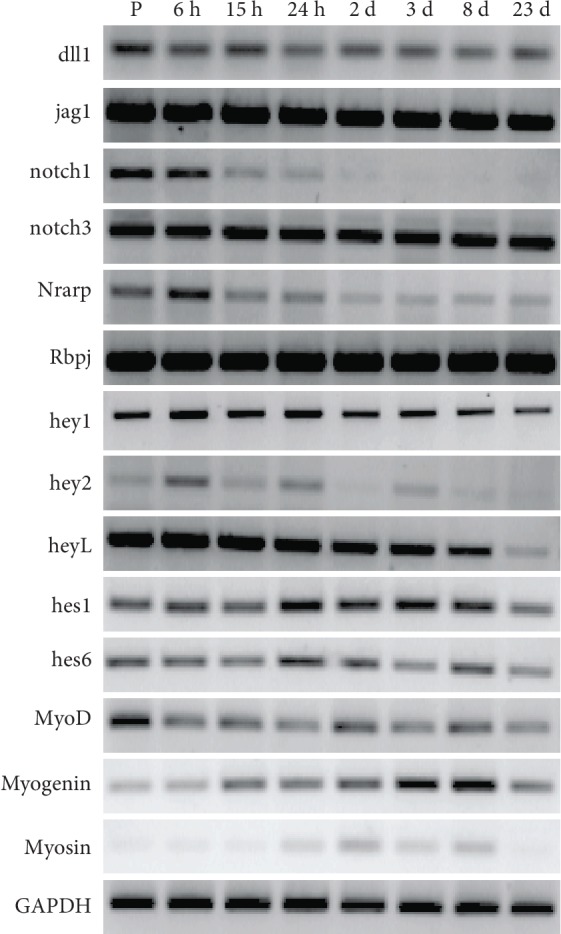
Expression of notch pathway genes in proliferating cells and in myospheres at different stages by RT-PCR. The figure summarizes experiments performed more than three times.

Notch activity plays a central role in controlling the quiescence of SC [18, 19].  Figure 5 shows an RT-PCR profile of factors related to the notch pathway in myospheres at different stages of maturation and in proliferating C2C12 cells. It shows that the cells in myospheres display all the factors required for an operative notch pathway. Indeed, in a 3D culture environment that is ideally suited to the promotion of cell-cell contacts, the coexpression of the notch ligands, dll1 and jag1, with their receptors, notch1 and notch3, ensures that the notch pathway can be activated. The constant presence of the Rbpj mediator guarantees the intracellular prosecution of the signal that switches on hes/hey downstream genes, which in turn inhibit myogenic factor expression and differentiation of the cells [22, 23].

According to their expression level, notch pathway factors in myospheres can be divided into two groups. In the first, which includes notch3, hey1, hes1, hes6, and Rbpj as well as the activators dll1 and jag1, the level of transcription remains approximately constant throughout culture time. The second group, which includes notch1, hey2, heyL, and Nrarp, shows a downregulated expression which reaches its minimum when differentiation terminates.

To investigate whether cell isolation affects the expression of notch genes in SC [6, 7], we checked their level of expression before and after enzymatic dissociation of mature myospheres.

**Figure 6:**
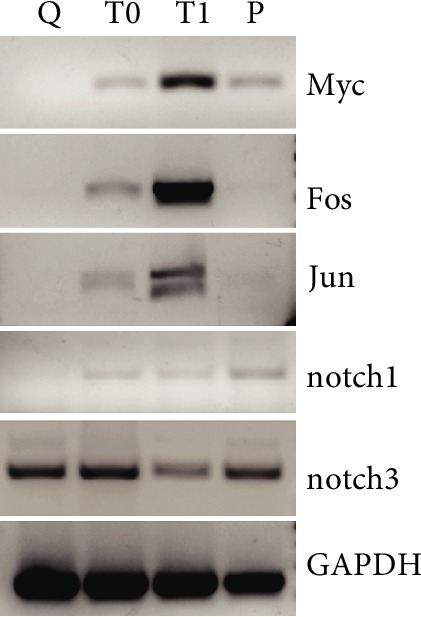
Dissociation of myospheres induces changes in the transcription pattern of the cells. Q: RNA extraction performed from mature myospheres; T0: cells processed for RNA extraction after myosphere dissociation was completed; T1: cells maintained in suspension in the culture medium at 37°C for 1 h after myosphere dissociation; P: cells from a monolayer.

Figure 6 shows that notch1 expression resumes after dissociation and accompanies that of notch3.

Since mature myospheres express only notch3 and display a low level of notch activation, we asked ourselves whether this level of hes/hey gene expression controlled the quiescent state of the cells.

**Figure 7:**
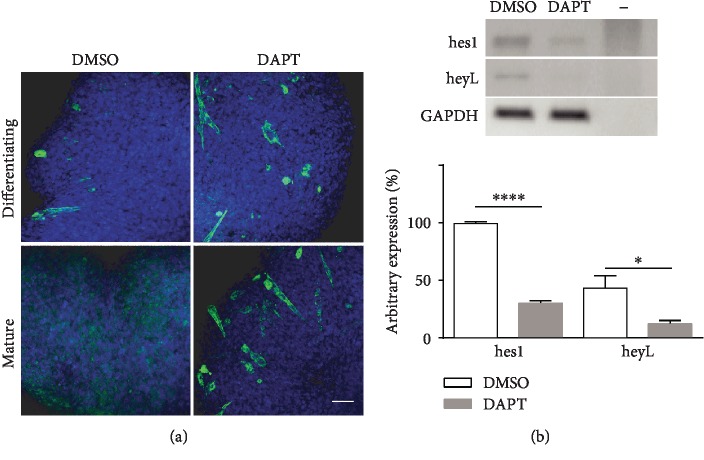
Differentiation resumes in mature myospheres following inhibition of the notch pathway. (a) Confocal image of whole-mount myosin immunofluorescence on differentiating (9 days) and mature (25 days) myospheres treated for 5 days with DAPT and respective DMSO controls. The effect of notch pathway inhibition is not particularly evident in differentiating myospheres because syncytia continue to leave the myospheres as they are formed. By contrast, DAPT resumes differentiation in mature myospheres, which retain the syncytia. Scale bar: 50 *μ*m (b) RT-PCR showing the change of hes1 and heyL expression in mature myospheres in response to 5 *μ*M DAPT treatment. Below the densitometric analysis of hes1 and heyL bands. ^∗^0.01 < *p* < 0.05 and ^∗∗∗∗^*p* < 0.0001.

For this purpose, we used the *γ*-secretase inhibitor DAPT, which inhibits notch signalling by blocking the generation of NICD from all notch paralogs. In preliminary experiments, we treated mature myospheres for 5 days with 5 *μ*M DAPT and evaluated the expression levels of hes1 and heyL by RT-PCR. Compared with control DMSO-treated aggregates, DAPT reduced the expression of hes1 and heyL, respectively, by 75% and 79% (Figure 7).

The cells of mature myospheres treated with DAPT exit quiescence and differentiate. Indeed, whole-mount immunofluorescences performed with MF20 anti-myosin antibody on DAPT and control DMSO-treated mature myospheres demonstrated that the generation of new syncytia resumed following inhibition of notch signalling, (Figure 7(a)) with confocal microscopy also revealing the presence of elongated myotubes.

## 3. Discussion

Our work complements that published in other studies that used myosphere cultures maintained within a chemically defined media reach of growth factors. Such myospheres promote the proliferation of myogenic stem cells that conserve their differentiation potential [8–15].

The data collected in these studies, together with our finding showing that myospheres grown in a medium with 10% FCS contain cells that are in a state of reversible quiescence having the morphology of cells whose metabolic activity is reduced, show that stem cells can be directed towards proliferation or reversible quiescence while preserving their staminality merely by manipulating the growth factor component of the niche. Furthermore, this highlights the ductility of the niche that emerges in 3D cultures. This observation may be further exploited experimentally to investigate how the addition of different cell types, matrix components, or soluble factors in the culture affects the niche as well as the behaviour of SC. Another advantage is that the high cellular density allows the cells to be analyzed as they are in the intact niche, without the need for the isolation or concentration procedures required when dealing with SC in vivo, which are dispersed on muscle fibers. Quiescent stem cells react to the loss of interactions with the niche that accompanies any isolation procedure by changing their metabolic and gene expression pattern [37]. Quiescence of the cells within the myospheres is proved by the G0/G1 DNA content and Ki67 expression. Differentiating cells are lost during the maturation of myospheres and the remaining cells express markers widely used to recognize SC in vivo. Individually, none of these markers indicates the quiescent or proliferating state of the cells, but when combined, their expression is indicative of quiescence.

CD34 is the factor that we analyzed in most detail. Although this factor is not a specific marker of myogenic cells *in vivo*, it is useful within the context of C2C12 myospheres because it is expressed in quiescent cells alone and rapidly disappears after activation. In particular, quiescent and activated SC *in vivo* produce differently spliced isoforms of CD34 mRNA, i.e., full-length (fl) and truncated (tk) [31, 38, 39], a pattern of expression that is reproduced in myospheres and the monolayer of C2C12 cells. The detection of the CD34 protein, which does not start before the 7th–9th day of culture, may indicate that the cells reach a state of complete quiescence only at this point. In this regard, Coller et al. reported that the transcriptional signature of fibroblasts induced to enter quiescence initially differs depending on how this state is induced, i.e., by mitogen withdrawal, loss of adhesion, or contact inhibition. Fibroblasts converge on a more common pattern of quiescent gene expression only many days later [2]. The expression of CD34 may mark a similar achievement for the cells in myospheres. The fact that the size of C2C12 cells in myospheres is smaller than that of those that proliferate in a monolayer points to a deeper state of quiescence, which is in agreement with the smaller size and lower mitochondrial and metabolic activity demonstrated in deeply quiescent satellite cells in skeletal muscle (G_0_ cells) [28]. The ease with which exchanges with the environment occur may also influence the activity of the niche and affect differentiation. The exchange of substances with the environment is less efficient in myospheres than in monolayer cultures, although the lack of hif-1*α* accumulation in our cells indicates that at least oxygen diffusion is correct (Figure 4). When Marquette et al. cultured, in the same medium as those used by us, C2C12 aggregates in the rotating cell culture system, which is known to induce a higher mass transfer of nutrients, they reported better differentiation rates in the absence of proliferation in their stationary control cultures corresponding to those that we used [35].

Furthermore, in keeping with our results, Marquette et al. [35] found that C2C12 myoblasts in such cultures fuse without proliferating beforehand, a behaviour that was more recently shown not to be abnormal even *in vivo*, as SC have been found to fuse more frequently than was previously believed with uninjured fiber without previous proliferation [40].

Notch signalling plays a primary role in directing SC toward proliferation, differentiation, or quiescence, and its expression and activity displays a high degree of sensitivity to manipulation of the niche [7]. When aggregation is completed, notch1 expression is downregulated and myogenic cells express only the notch3 isoform. If a dissociation procedure activates the cells, the expression of notch1 soon resumes and accompanies that of notch3. The pattern of hes/hey expression in mature aggregates diverges from that reported in SC that have been freshly sorted from muscle, which appear to express higher levels of hes/hey genes in the quiescent state than when proliferating [18, 19]. The absence of notch1 signalling in myospheres may explain such differences. Indeed, notch3 alone is considered a weak activator of hes/hey genes [41], whose expression in mature myospheres is consequently lower than that of proliferating C2C12 cells. The pattern of expression of notch3 and of notch1 in myospheres is also consistent with studies that report a role of notch3 in maintaining the homeostasis of stem cells by guarding cell cycle access [42, 43].

In conclusion, our data suggest that myogenic cells that self-assemble in three-dimensional cultures in 10% FCS form a system that preserves the quiescence and the staminality of the cells. Taking the opportunity to examine these cells without any previous manipulation, we show that in this system, the cells express notch3 mRNA and fail to express notch1. Consequently, in contrast to previous findings [18, 19], we show that the activity of notch downstream factors in quiescent C2C12 cells is lower than that in proliferating cells.

We believe that C2C12 3D sphere cultures may be effectively used as a model to address unresolved questions in stem cell biology, such as how cell composition, matrix components, or diffusible factors affect the biological activity of the niche. Notably, C2C12 3D sphere cultures may be a good tool to promote skeletal muscle repair by delivering such cells to the damaged tissue.

## 4. Materials and Methods

C2C12 cells between the 4^th^ and the 20^th^ passage were maintained in a monolayer in DMEM glutamax (EuroClone, Pero (MI), Italy) supplemented with 10% FCS (EuroClone) and 50 *μ*g/ml gentamicin in a humidified atmosphere of 5% CO_2_ at 37°C and passaged at 70-80% confluence.

To generate aggregates, cells were grown to 80-85% confluence, detached enzymatically, and seeded at a density of 3 − 4 × 10^5^/ml on 90 mm bacterial dishes in the same culture medium as that used to grow them in a monolayer with the addition of 0.3% methylcellulose (Sigma-Aldrich, St. Louis, MO, USA, Cat. 09967). In these conditions, cells do not adhere to plastic and join together in aggregates of various sizes (http://spherogenex.de) [44]. The medium was changed every 2-3 days.

### 4.1. Cell Count

3D cultures contain aggregates of different sizes that do not disperse homogeneously in the plate. Consequently, sampling problems arise when aliquots of the medium are being collected. For this reason, in order to evaluate the number of cells remaining in aggregates after differentiation, we counted the cells obtained after trypsin dissociation of entire dishes.

### 4.2. Inhibition of the Notch Pathway

The *γ*-secretase inhibitor DAPT (Sigma Aldrich) was dissolved in DMSO and used at a concentration of 5 *μ*M. The drug was added for 5 days on the 4^th^ day of culture (differentiating aggregates) or on the 20^th^ day (mature aggregates). In the latter case, the drug was added after the end of differentiation as indicated by the disappearance of a halo of dying cells surrounding the aggregates while cells differentiate. Culture medium with DAPT or DMSO was replaced every 2 days.

### 4.3. Frozen and Paraffin Section Preparation

Aggregates were fixed for 2 h with 4% paraformaldehyde (Sigma-Aldrich), at 4°C, washed with PBS, and stored at 4°C. Frozen sections (7 *μ*m) were prepared after embedding of aggregates in OCT (Tissue*-*Tek, Sakura Finetek USA). Sections were incubated with primary antibodies directed against Ki67 (Santa Cruz Biotechnology, Inc., Heidelberg, Germany, SC7846, 1 : 100), laminin (Sigma L939, 1 : 100), CD34 (Santa Cruz Cat. SC9095, 1 : 100), and MF20 anti-sarcomeric myosin heavy chain (1 : 5 diluted hybridoma medium). Fluorescent images were acquired using an epifluorescence Zeiss Axioskop 2 Plus microscope (Carl Zeiss, Oberkochen, Germany) or a Leica Leitz DMRB microscope fitted with a DFC300FX camera (Leica, Wetzlar, Germany). For haematoxylin and eosin (H&E) staining, fixed aggregates were dehydrated, embedded in paraffin wax, and cut into 5 *μ*m sections. Images were assembled using Adobe Illustrator or ImageJ.

### 4.4. Whole-Mount Immunofluorescence

Aggregates were fixed in cold (-20°C) methanol-acetone 1 : 1 (*v*/*v*) for 20 minutes and washed with PBS. They were then incubated, by shaking with MF20 anti-myosin antibody, (from hybridoma culture medium, diluted 1 : 5) at 4°C for 20 h. Washed aggregates were incubated with fluorescein-labelled secondary antibodies for 2 h at room temperature. Nuclei were stained with TO-PRO-3 (0.2 mg/ml, Molecular Probes, Molecular Probes Europe, Rijnsburgerweg, The Netherlands). Samples were observed by confocal microscopy with a Leica TCS SP2 (Leica, Wetzlar, Germany).

### 4.5. Fusion Index Quantification

The fusion index was measured in cells grown for 72 h in DM after reaching confluence and stained with MF20 anti-myosin antibody. This index was calculated as the ratio of the number of nuclei in myotubes with 3 or more nuclei versus the total number of nuclei in the field.

In detail, the comparison of the fusion index between cells from mature aggregates and original cells was performed as follows: fresh C2C12 cells were grown to 70-80% confluence in 6 different 150 mm dishes. The cells from such dishes were then dissociated and transferred each to a different 90 mm dish in nonadherent conditions to generate the myospheres, which were then left to mature. Three days after the disappearance of the halo, the myospheres (not counted) from each of the 90 mm dishes were dissociated and a portion of the cells obtained was plated in three 35 mm dishes (30000 cells/dish) and compared for their capacity of differentiation with fresh control cells plated in the same conditions in parallel in five 35 mm dishes.

### 4.6. Cell Cycle Analysis

Cells derived from trypsin dissociation of the aggregates were fixed in cold 80% ethanol, washed, and incubated at 37°C for 3 h in PBS containing 1% Triton X-100, 50 *μ*g/ml propidium iodide, and 100 *μ*g/ml DNase-free RNase A (Sigma-Aldrich). Flow cytometric analysis was performed on an Epics XL (Beckman Coulter, Fullerton, CA, USA) using Expo 32 software for acquisition and FCS Express 4 for analysis. Doublets were discriminated by means of gating.

### 4.7. BrdU Labelling

Cells obtained by the trypsinization of mature aggregates were plated at a density of 8 − 10 × 10^3^/ml in 35 mm dishes. After 24 h, the cells were pulse-labelled with 10 *μ*M BrdU (Sigma-Aldrich) in growth medium for 4 h. At 90-100% confluence, the growth medium was changed to DMEM + 2%HS for 4-5 days with daily changes. After fixation, cells were stained with anti-BrdU (Sigma B2531) and MF20 anti-myosin antibody.

### 4.8. Halo Characterization

To characterize the fate of the cells lost by myospheres during the maturation process and their subsequent fate, we examined the material released from myospheres by day 9 after 2 days of culture. Myospheres with their medium were transferred to a tube and left to settle by gravity. The spent medium was then removed and centrifuged at 1000 rpm, and the pellet containing released cells and cellular fragments was resuspended without fixation in PBS for microscopical examination after staining with 1 *μ*M Hoechst 33342, 2.5 *μ*g/ml propidium iodide and annexin V, and Alexa Fluor™ 488 conjugate (Thermo Fisher Scientific A13201), all from 100x solutions. Samples were stained in ice for 5 min and discarded after a further 10 min because propidium iodide is no longer specific for dead cells after this time.

### 4.9. Semiquantitative RT-PCR

Total RNA was extracted by using TRI Reagent (Sigma T9424) according to the manufacturer's protocol. A 1 *μ*g aliquot of total RNA was treated with DNase-I (New England Biolabs, EuroClone, Pero (MI), Italy) and reverse transcribed into first-strand cDNA using M-MLV reverse transcriptase (M-1705, Promega, Madison, WI, USA) and random hexamer primers (Invitrogen, Thermo Fisher Scientific Inc., Waltham, MA USA). Cycling parameters were 94°C/20 s, 58°C/20 s, and 72°C/20 s for 31 cycles. PCR products were run on 2% agarose gels. PCR was performed with the following primers: caveolin-1 (5′-GCACACCAAGGAGATTGACC-3′; 5′-GAATGGCAAAGTAAATGCCC-3'), jag1 (5′-*CCCCCTGAGTCTTCTGCTC-3*′; 5′-*GTGACGCGGGACTGATACTC-3*′, M-Cad (5′-ATGATGGCTCTGTACCAGC-3′; 5′-AAGACTACGACCCAGAAGAC-3′), c-Met (5′-TCCTGACATCCATCTCCACC-3′; 5′-GCATGAAGCGACCTTCTGAC-3′), Pax7 (5′-CCGTGTTTCTCATGGTTGTG-3′; 5′-GAGCACTCGGCTAATCGAAC-3′), MyoD (5′-AGCACTACAGTGGCG ACTCA-3′; 5′-GCTCCACTATGCTGGACAGG-3′), myosin (5′-ACAAGCTGCGGGTGAAGAGC-3′; 5′-CAGGACAGTGACA AAGAACG-3′), myogenin (5′-CTACAGGCCTTGCTCAGCTC-3′; 5′-AGATTGTGGGCGTCTGTAGG-3′), c-Myc (5′-CGGACACACAACGTCTTGGAA-3′; 5′-AGGATGTAGGCGGTGGCTTTT-3′), c-Jun (5′-CTGCATGGACCTAACATTCG-3′; 5′-GCTTTCACCCTAGTATATTGGG-3′), c-Fos (5′-TGTTGTTCCTAGTGACACC-3′; 5′-ACATTCAGACCACCTCG-3′), notch1 (5′-TGCC TGTGCACACCATTCTGC-3′; 5′-CAATCAGAGATGTTGGAATGC-3′), notch3 (5′-TGCCAGAGTTCAGTGGTGG-3′; 5′-CACAGGCAAATCGGCCATC-3′), dll1 (5′-CCCTGGCAGACAGATTGG-3′; 5′-ACAGAGGGGAGAAGATGTGC-3′), hes1 (5′-GCCAATTTGCCTTTCTCATC-3′; 5′-GAGAGGTGGGCTAGGGACTT-3′), hes6 (5′-CCCTAGAGCTCTGGATGGTG-3′; 5′-GCGCAACTGTGTTACAAACG-3′), hey1 (5′-TCA GCGTGGGGAATCTTAAC-3′; 5′-GATTCAGGGCACAGACACCT-3′), hey2 (5′-TGGAAAAGGAAACGCCAT A-3′; 5′-ATCTGCAGCCTGACACATTG-3′), heyL (5′-GCGATTGAAGTCCCCAG ATA-3′; 5′-ACTGGGGTCACCAGACTG AG-3′), Rbpj (5′-GGTCCCAGACATTTCTGCAT-3′; 5′-GGAGTTGGCTCTGAGAATCG-3′), GAPDH (5′-TCCTGACATCCATCTCCACC-3′; 5′-GCATGAAGCGACCTTCTGAC-3′). Primers used to distinguish full-length and truncated forms of CD34 transcripts were 5′-AGCACAGAACTTCCCAGCAA-3′ in exons 5/6 and 5′-CCTCCACCATTCTCCGTGTA-3′ in exon 8 (Beauchamp et al., 2000), which amplify, respectively, a fragment of 260 and 416 bp, as the truncated form includes an extra exon with a stop codon that arrests translation.

### 4.10. Immunoblot Analysis

Cells were lysed by sonication in ice-cold RIPA buffer (20 mM Tris-HCl pH 7.6 containing 150 mM NaCl, 2 mM EDTA, 1% NP40, 0.5% Na-deoxycholate) supplemented with protease inhibitor cocktail (Sigma-Aldrich P2714). Equal amounts of proteins were resolved on 8-10% SDS polyacrylamide gel. After being transferred onto PVDF membranes (Amersham GE Healthcare, Buckinghamshire, UK), proteins of interest were detected by using anti-CD34 (sc-9095 Santa Cruz, 1 : 500), anti-myosin (MF20 1 : 50), anti-MyoD (sc-377460 Santa Cruz, 1 : 1000), anti-myogenin (sc-12732 Santa Cruz, 1 : 300), anti-hif-1*α* (NB100-105 Novus Biologicals, Segrate, Italy, 1 : 1000), and anti-caveolin-1 (ab17052 Abcam, Cambridge, UK 1 : 1000) antibodies and anti-*α*-tubulin (T5168, Sigma, 1 : 2000) or anti-GAPDH (Santa Cruz, sc-25778, 1 : 1000) antibodies as a normalization control.

### 4.11. Statistical Analysis

Quantitative data of at least three independent experiments are presented as means ± SEM. Statistical analysis was performed by means of Graphpad Prism Software (La Jolla, CA, USA) using paired Student's *t*-tests to determine significance (^∗^0.01 < *p* < 0.05; ^∗∗^0.001 < *p* < 0.01; ^∗∗∗^0.0001 < *p* < 0.001; ^∗∗∗∗^*p* < 0.0001).

## Data Availability

All data used to support the findings of this study are included within the article.

## Supplementary Materials

Supplementary Materials Supplementary Fig. 1: genesis of the halo. Cells released from the myospheres between the seventh and ninth days of culture. Once the cells are isolated in the medium, they enter apoptosis. The sample shown in the figure contains cells at all stages of apoptosis since new cells are continuously released from myospheres. The new cells join those previously released and their debris, trapped by the released DNA that also induces their attachment to the microspheres. In fact, these aggregates are rapidly dissolved by treatment with DNase. (a) phase-contrast image; (b) Hoechst 33342 staining, labeling all nuclei; (c) propidium iodide staining, labeling dead cell nuclei; (d) annexin V staining. Annexin V binds to phosphatidylserine residues translocated to the external face of the plasma membrane. This is an early event in apoptosis. Bar = 50 *μ*m.
