# Supplementary material for: A Three-Dimensional Culture Model of Reversibly Quiescent Myogenic Cells
Source: Stem Cells Int. 2019 Nov 11;2019:7548160. doi: 10.1155/2019/7548160 (PMC6885280; doi:10.1155/2019/7548160)
Supplement: Supplementary Materials — Supplementary Fig. 1: genesis of the halo. Cells released from the myospheres between the seventh and ninth days of culture. Once the cells are isolated in the medium, they enter apoptosis. The sample shown in the figure contains cells at all stages of apoptosis since new cells are continuously released from myospheres. The new cells join those previously released and their debris, trapped by the released DNA that also induces their attachment to the microspheres. In fact, these aggregates are rapidly dissolved by treatment with DNase. (a) phase-contrast image; (b) Hoechst 33342 staining, labeling all nuclei; (c) propidium iodide staining, labeling dead cell nuclei; (d) annexin V staining. Annexin V binds to phosphatidylserine residues translocated to the external face of the plasma membrane. This is an early event in apoptosis. Bar = 50 μm. [file 7548160.f1.pdf]

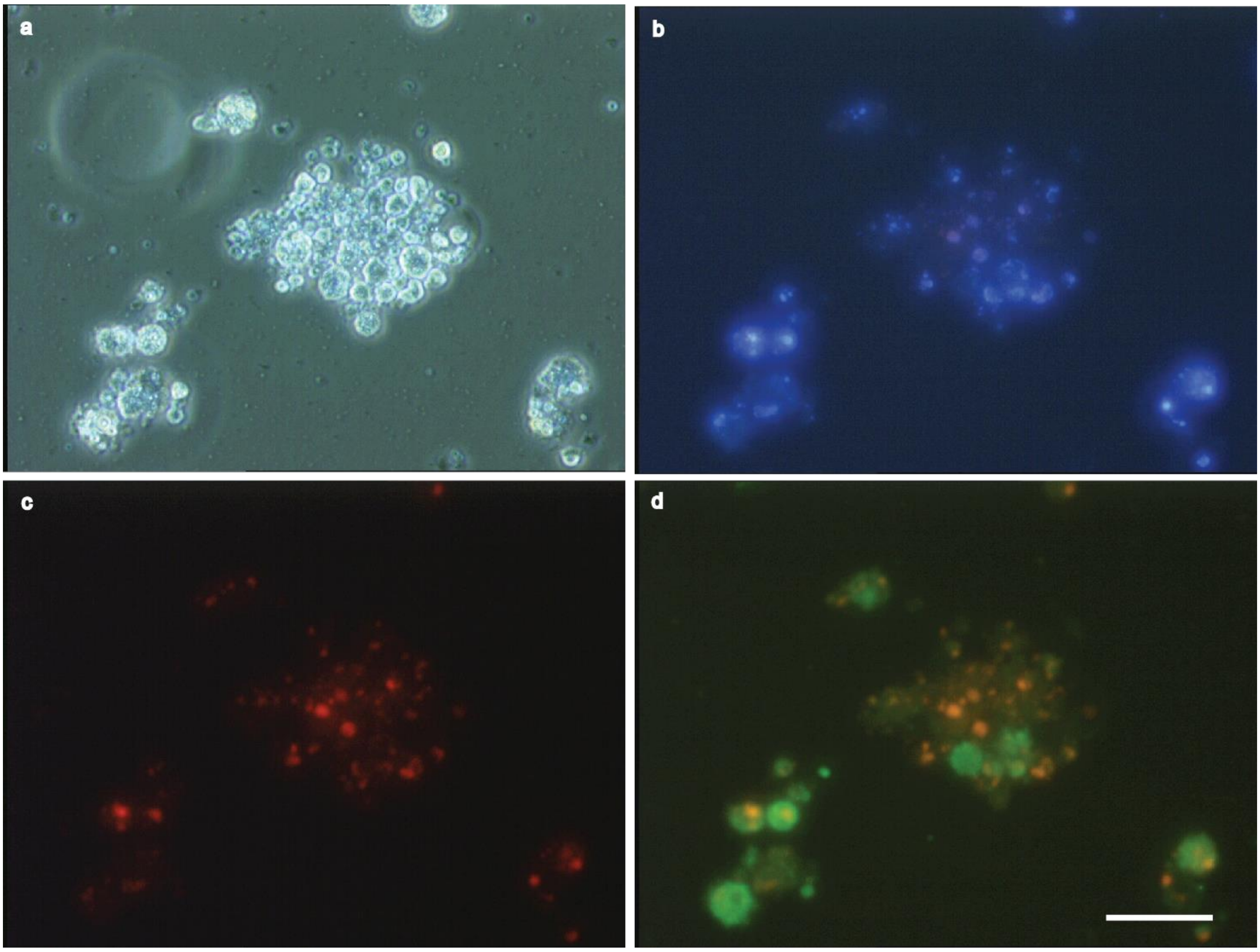

Supplementary Fig. 1. Genesis of the Halo.
Cells released from the myospheres between the seventh and ninth day of culture. Once the cells are isolated in the medium enter in apoptosis. The sample contains cells at all stages of apoptosis since they are continuously released from myospheres.
a, phase-contrast image; **b**, Hoechst 33342 staining, labeling all nuclei; **c**, Propidium Iodide staining, labeling dead cells nuclei; **d**, Annexin V staining. Annexin V binds to phosphatidylserine residues translocated to the external face of the plasma membrane. This is an early event in apoptosis. These aggregates stick to myospheres but are quickly dissolved with DNase treatment.
Bar = 50µm
